# Pulse sharpness as a quantitative index of vascular aging

**DOI:** 10.1038/s41598-021-99315-8

**Published:** 2021-10-06

**Authors:** Jang-Han Bae, Young Ju Jeon

**Affiliations:** Digital Health Research Division, Korea Institute of Oriental Medicine, 1672 Yuseong-daero, Yuseong-gu, Daejeon, Republic of Korea

**Keywords:** Computational biology and bioinformatics, Biomarkers, Cardiology, Health care, Risk factors, Engineering, Mathematics and computing

## Abstract

The aim of this study was to develop a robust algorithm to quantify pulse sharpness that can complement the limitations of radial augmentation index (rAIx) and explore the role of this quantitative sharpness index in reflecting vascular aging or arterial stiffness. The pulse sharpness index (PSI) was developed by combining the end point angle and virtual height, and 528 radial pulses were analyzed. The PSI could be uniformly applied to various waveform morphologies, even those with no or vague tidal waves, unlike the rAIx. Significant sex differences were identified in the rAIx and PSI (P < 0.01 for both), and significant age-dependent decreases in the PSI were observed (P < 0.01). In addition, the PSI and age were correlated (r = − 0.550) at least as strong as the rAIx and age (r = 0.532), and the PSI had a significant negative correlation with arterial stiffness (r = − 0.700). Furthermore, the multiple linear regression model for arterial stiffness using the PSI, age, sex and heart rate showed the excellent performance (cross-validated R^2^ = 0.701), and the PSI was found to have the greatest influence on arterial stiffness. This study confirmed that the PSI could be a quantitative index of vascular aging and has potential for use in inferring arterial stiffness with an advantage over the rAIx.

## Introduction

As age increases, the elasticity of arterial vessels decreases, and the inner diameter widens due to changes in the mechanical properties of blood vessels and the cardiovascular system^1,2^. A decrease in elasticity leads to the stiffness of arterial vessels increasing and the reflected wave returning quickly^3,4^, and abnormalities in proximal aortic diameter are responsible for the abnormal aortic pressure-flow relationship^1,5^. This phenomenon increases arterial pressure, which elevates the risk of cardiovascular disease (CVD), including hypertension^6,7^. Therefore, examining these age-related changes in arterial stiffness and appropriate indicators reflecting the stiffness caused by cardiovascular aging are important to prevent risk factors for CVD^8^.

The pulse wave velocity (PWV), one of the representative indicators of arterial stiffness, is the speed at which a blood pressure pulse wave propagates through the arterial system. There is a distance measurement error caused by assuming a straight arterial segment, and in the most frequently used carotid-femoral PWV, the carotid and femoral pulse waves traveling in opposite directions cause overestimation of the PWV^6^.

Another measure of arterial stiffness is the augmentation index (AIx), which is the ratio of the height of the peak above the shoulder of the wave to the pulse pressure^9^. The AIx is easier to measure and less time consuming than the PWV; additionally, the AIx enables observation of the reflective properties of the arteries^10^. The AIx increases with age and blood pressure, which has been explained by the fact that wave reflection increases with age^3,11^. In addition, AIx is known as an independent risk factor for CVD, including coronary artery disease^12,13^, heart failure^14^, hypertension^15^, diabetic retinopathy^16^ and atherosclerosis^17^.

An important step in calculating the AIx is exact identification of the shoulder point on the waveform^18^. At this time, because a close correlation between the aortic AIx and radial AIx (rAIx) has been demonstrated, similar information on the reflection of the central pressure wave can be obtained directly from the radial pulse waveform^19^. In the case of the radial or type C waveform, the algorithm based on the second negative-to-positive zero-crossing of the fourth derivative pulse waveform has been established to determine the AIx and has been widely used. Recently, because numerical differentiation could amplify noise, especially at high frequencies, a robust algorithm based on a B-spline wavelet or only first- and second-order derivatives of the waveforms have been proposed for improved accuracy and reduced noise^18,20^. However, there are other inherent limitations in the calculation of AIx aside from algorithm dependency. If the tidal wave in the radial pulse becomes vague, the inflected shoulder point becomes difficult to extract accurately; additionally, in the case of the pulse waveform with only two peaks and without a tidal wave, the AIx cannot be calculated or set to zero^21^. The most appropriate mathematical expression of the AIx is unclear; in other words, the AIx is an incomplete index because the method for calculating the AIx does not consider various pulse morphologies^8,22^. Furthermore, an average pulse morphology variability of 7.2% near the rAIx was observed, as determined according to the intra-class distance within a single-period pulse in a previous study, and the variabilities of several pulses were greater than 15%^23^. These values are now low, and another index that is robust to the morphology variability is needed.

The sharpness of the pulse wave is a representative characteristic in pulse waveform analysis. Because it is well known that blunter waveforms are generally found in older people, whereas sharper pulse waveforms are found in younger people, pulse sharpness could reflect age-related characteristics, such as vascular aging or arterial stiffness, as well as the AIx. In addition, as the average pulse morphology variability near the peak point of the percussive wave was less than only 2% in a previous study, pulse sharpness could be an index with low variability^23^. However, despite these intuitive considerations of pulse sharpness, few studies have been explored the availability of sharpness as an indicator of vascular aging or arterial stiffness, unlike the AIx. In addition, a formally accepted algorithm for detecting sharpness has not been reported. Although a method for finding both end points of the width of a percussive wave based on determining 2/3 the pulse height and calculating the distance, defined as w, between the two end points has been proposed for calculating the pulse apex angle, it is difficult to derive an accurate result if a tidal wave is generated at a higher position than 2/3 the pulse height^24–27^. In addition, in previous studies, the apex angle was calculated based on coordinates using four points before and after the pulse peak^28^ and on the law of cosines using two neighboring fiducial points^29^, but these methods were applicable only to the specific shape of the pulse waveform.

Therefore, an objective and easily computable index reflecting pulse sharpness that can be uniformly applied to various waveform morphologies is needed. Furthermore, if the possibility for the clinical usefulness of pulse sharpness is demonstrated, the limitations of the AIx could be overcome. The aim of this study was to develop a robust algorithm to quantify pulse sharpness that can complement the limitations of rAIx and explore the role of this quantitative sharpness index in reflecting vascular aging or arterial stiffness.

## Results

A comparison of the PSI, the first proposed index in this study, according to pulse waveform samples with differences in pulse sharpness was performed, and details of the results are provided in Supplementary Fig. S1. The PSI could be uniformly applied to various pulse morphologies, including waveforms that are difficult or too obscure to use in calculating the rAIx or pulse apex angle using w accurately.

### Mean difference analysis by sex and age

Table 1 shows the baseline characteristics, including the distribution of sex, age, heart rate (HR), systolic blood pressure (SBP), diastolic blood pressure (DBP), pulse pressure, height and weight and the sex differences in the rAIx and PSI in each age group with the mean and standard deviation (SD). The rAIx in men was significantly lower than that in women in the entire group and in the groups of subjects in their 20 s, 40 s (P < 0.01), and 30 s (P < 0.05). The PSI in men was significantly higher than that in women in the entire group and in those from their 20 s to 40 s (P < 0.01). Both indices showed no significant sex difference in those over 50 years old.

**Table 1:** Baseline characteristics and sex differences in the rAIx and PSI in each age group (mean ± SD).

	Men	Women	P-value
Number of subjects	137	367	
Age (year)	35.81 ± 10.85	39.73 ± 11.89	< 0.01**
HR (beats per minute)	69.57 ± 9.21	70.57 ± 8.64	0.26
SBP (mmHg)	118.71 ± 12.18	113.52 ± 14.34	< 0.01**
DBP (mmHg)	76.85 ± 10.72	74.11 ± 10.05	< 0.01**
Pulse pressure (mmHg)	41.86 ± 10.76	39.41 ± 10.03	< 0.05*
Height (cm)	173.40 ± 6.43	158.97 ± 8.76	< 0.01**
Weight (kg)	72.60 ± 11.31	57.64 ± 8.52	< 0.01**
**rAIx (%)**			
Entire group	64.61 ± 22.26	77.91 ± 21.00	< 0.01**
20 s	47.50 ± 14.90	59.47 ± 15.16	< 0.01**
30 s	69.01 ± 20.42	83.65 ± 19.33	< 0.05*
40 s	75.03 ± 19.66	86.34 ± 18.61	< 0.01**
Over 50 years	87.01 ± 19.76	83.46 ± 17.01	0.59
**PSI (degree)**^**−1**^			
Entire group	13.76 ± 3.21	11.38 ± 3.28	< 0.01**
20 s	15.92 ± 2.71	14.11 ± 2.94	< 0.01**
30 s	13.20 ± 2.79	11.44 ± 3.56	< 0.01**
40 s	12.75 ± 2.86	10.09 ± 2.37	< 0.01**
Over 50 years	9.72 ± 2.46	9.73 ± 2.36	0.98

*P < 0.05; **P < 0.01.

In the case of height and weight, there were no data in one clinical trial (IRB No. KOMCIRB-2014-70), so the values were calculated only from the data of the other five clinical trials.

**Figure 1:**
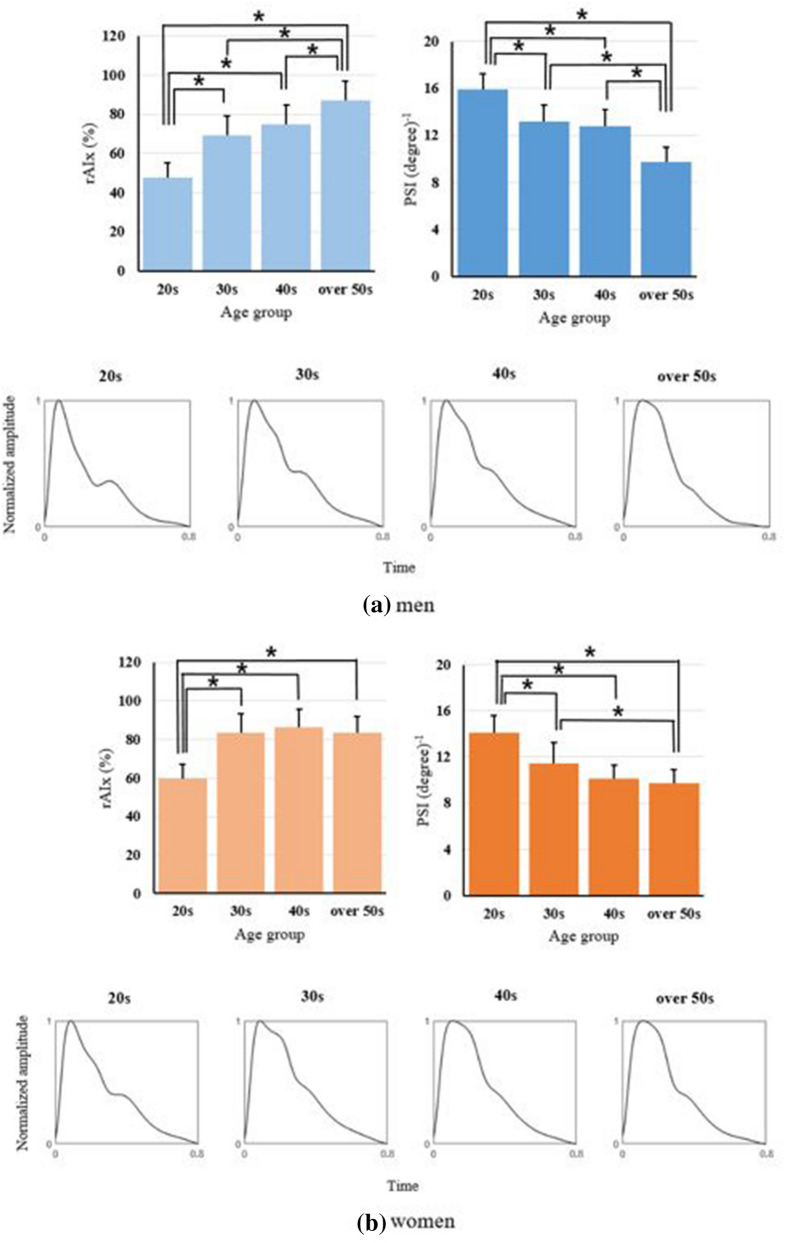
Age-related changes in the radial augmentation index (rAIx) and pulse sharpness index (PSI) according to (**a**) men and (**b**) women. The first row indicates the results of the one-way ANOVA in each age group, and the second row indicates the morphologies of ensemble average pulse waveforms in each age group. *P < 0.05.

The results of one-way ANOVA for observing the age-related changes in each age group according to sex are presented in Fig. 1 with the morphologies of the ensemble average pulse waveforms in each age group. Both the rAIx and PSI showed a significant difference according to age (P < 0.01). The rAIx was significantly lower in those in their 20 s than in those in the other age groups regardless of sex (P < 0.05); in men, the rAIx was significantly higher in those over 50 years old than in those in the other age groups (P < 0.05). The PSI showed significant results opposite those of the rAIx (P < 0.05 for those in their 20 s and over 50 years old), and the PSI of women in their 30 s was significantly different from that in women over 50 years old (P < 0.05). In addition, typical patterns of waveforms and age-related changes with respect to the rAIx and PSI were observed through the ensemble average pulse waveforms in each age group.

### Correlation and regression analysis according to age

**Figure 2:**
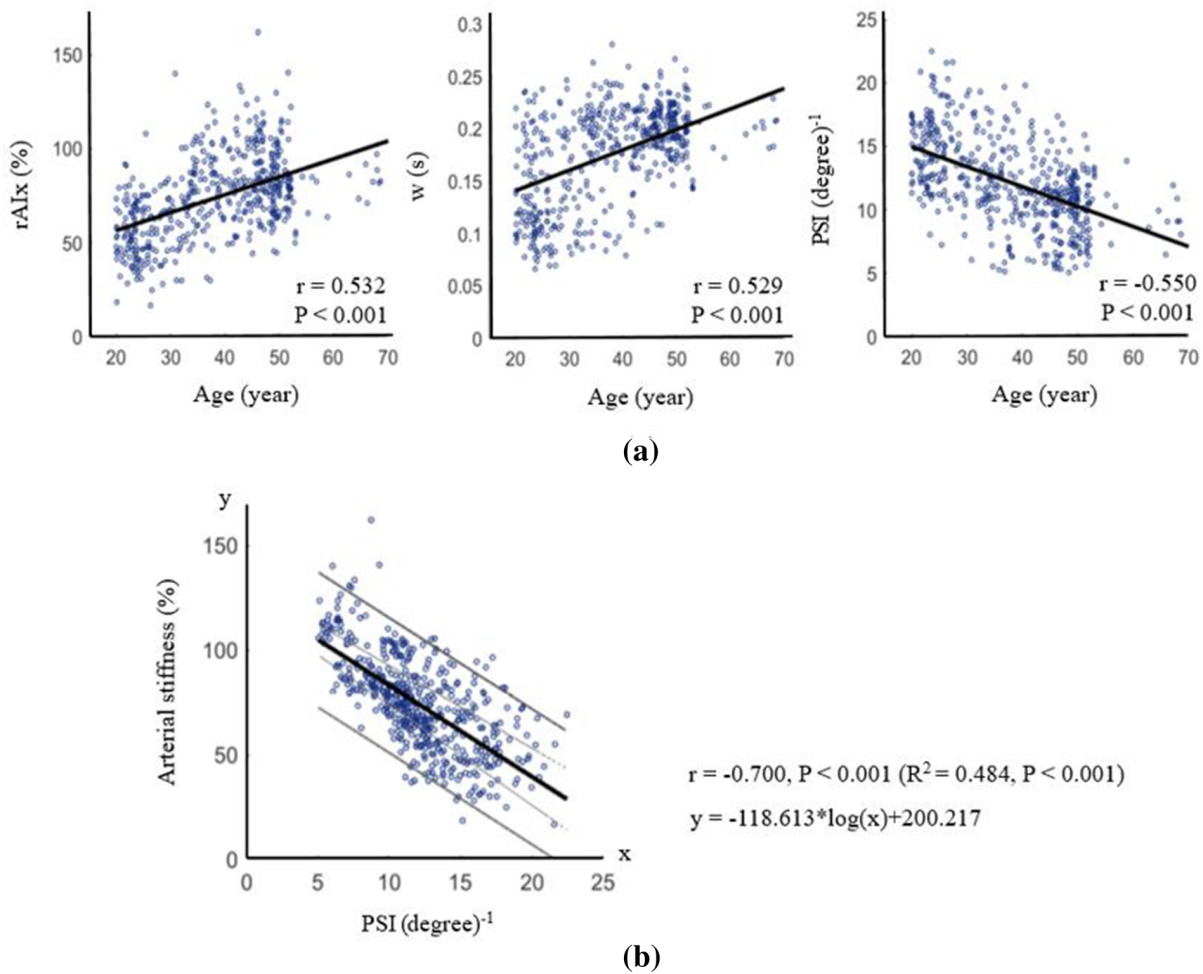
(**a**) Scatterplots of relationship between age and the radial augmentation index (rAIx), w and pulse sharpness index (PSI). (**b**) Scatterplots of relationship and the regression line between the PSI and arterial stiffness inferred by the rAIx. Narrow dotted lines represent the 95% confidence interval of the mean, and wide solid lines represent the 95% prediction interval for individual observations.

Figure 2a shows scatterplots of the relationship between age and various indices. Positive associations between age and the rAIx and between age and w were observed (correlation coefficient (r) = 0.532 and 0.529, respectively, P < 0.001 for all). On the other hand, a highly negative association between age and the PSI was observed (r = − 0.550, P < 0.001). Figure 2b shows a scatterplot of the relationship and the regression line between the PSI and arterial stiffness inferred by the rAIx. The PSI showed a strong and significant negative correlation with arterial stiffness (r = − 0.700, P < 0.001), and the stiffness could be approximated from the PSI using the appropriate regression equations (R-squared (R^2^) = 0.484, P < 0.001). Subsequently, scatterplots of the relationship and the regression line between rAIx, PSI and age were shown in Supplementary Fig. S2 for all available data classified by index examination SBP group 1 through 3. The rAIx showed an increasing pattern with aging and the PSI showed a decreasing pattern with aging in all three groups. In addition, the group with lower SBP generally showed higher PSI values.

Table 2 presents the multiple linear regression (MLR) results for the rAIx and PSI according to age and sex and for arterial stiffness inferred by the rAIx according to the PSI, age, sex and HR. Because the rAIx and PSI showed significant differences by sex, a term for sex was considered in creating the regression model as well as a term for age. MLR analysis revealed that the rAIx and PSI were significantly associated with age and sex (adjusted R^2^ = 0.320 and 0.357, respectively, P < 0.001 for all). In addition, a higher β value for age than sex was shown for both indices. Arterial stiffness was significantly associated with all four variables (adjusted R^2^ = 0.699, P < 0.001), and the model including age, sex and HR showed an increased correlation (r = 0.838) compared with that of the model considering the PSI alone (r = − 0.700). In addition, the absolute β value of the PSI for stiffness (β = − 0.601) was found to be greater than that of age (β = 0.167); on the other hand, sex did not appear to have a relatively strong effect.

**Table 2:** MLR analyses of the rAIx and PSI according to age and sex and of arterial stiffness, inferred by the rAIx, according to the PSI, age, sex and HR.

Index	Variable	β	t	P-value	Adjusted R^2^
rAIx	Age*	0.505	13.614	< 0.001	0.320
	Sex	0.200	5.403	< 0.001	
PSI	Age*	− 0.518	− 14.343	< 0.001	0.357
	Sex	− 0.241	− 6.684	< 0.001	
Arterial stiffness	PSI*	− 0.601	− 19.960	< 0.001	0.699
	Age*	0.167	5.757	< 0.001	
	Sex	0.084	3.274	0.001	
	HR	− 0.424	− 17.160	< 0.001	

*Tested by log transformation.

Table 3 presents the cross-validated R^2^, root mean square error (RMSE) and mean absolute error (MAE) for the training and test datasets calculated by tenfold cross-validation to evaluate the performance of the three simple linear regression models and an MLR model.

**Table 3:** Evaluation of the performance of the simple linear regression models and a MLR model based on tenfold cross-validation.

Model	Index	Variable	Dataset	Cross-validated R^2^	RMSE	MAE
Model 1	rAIx	Age	Training	0.283	0.846	0.669
			Test	0.283	0.848	0.673
Model 2	PSI	Age	Training	0.302	0.834	0.660
			Test	0.319	0.823	0.653
Model 3	Arterial stiffness	PSI	Training	0.484	0.717	0.563
			Test	0.486	0.710	0.563
Model 4	Arterial stiffness	PSI, age, sex, HR	Training	0.700	0.551	0.427
			Test	0.701	0.548	0.431

Model 1 for the rAIx according to age showed a relatively lower cross-validated R^2^ value and lower RMSE and MAE values than model 2 for the PSI according to age. In addition, the performance of model 3 for predicting arterial stiffness inferred by the rAIx based on the PSI was better than that of model 1. Furthermore, MLR model 4 for arterial stiffness according to the PSI, age, sex and HR showed the greatest cross-validated R^2^ of 0.701 in the test dataset, and it showed excellent performance when evaluated using the RMSE and MAE. Because the performance was not improved with the quadratic model, the results are not described.

## Discussion

In this study, the PSI was proposed by combining the end point angle (EPA) calculated based on the intersecting tangent method (ITM) focusing near the peak point regions and virtual height (VH) considering the overall shape of the pulse waveform. It was confirmed that this quantifiable index could be uniformly applied to various waveform morphologies even with no or vague tidal waves, from which it is difficult to calculate the rAIx accurately. In addition, even if a tidal wave was generated at a position higher than 2/3 the pulse height, leading to an incorrect pulse apex angle derived from w, the PSI could also be applied to these waveforms for determining the pulse sharpness accurately. In other words, the PSI could objectively represent the pulse sharpness of any type of waveform, unlike the rAIx or w. Nevertheless, obscure waveforms that could not be used to calculate the rAIx clearly were excluded from this study for comparison with the PSI, and analysis with recognition of the limitation of the rAIx was performed.

A comparison of the rAIx and PSI between men and women showed a significant difference in the entire group and in those from their 20 s to 40 s. The rAIx in women was significantly higher than that in men, which is consistent with the findings of previous studies^10,11,30^. It has been shown that reflected waves return faster in women than in men because women are generally shorter than men, which leads to a higher rAIx in women than men. In this study, the height in men was significantly higher than that in women as expected, which was considered to be one of the factors that rAIx and PSI are significantly different according to sex.

In addition, the smaller diameter of the radial artery and higher PWV in women could also affect the higher AIx in women^31^. The PSI was significantly higher in men than in women, which indicates that men have a sharper pulse wave than women. This seems to be explained by the similar phenomenon by which reflected waves affect the rAIx, even though the PSI derived from percussive waves is affected by reflected waves slightly later in time than the rAIx.

A significant age-dependent increase in the rAIx and a significant age-dependent decrease in the PSI were observed in those from their 20 s to 40 s regardless of sex. For example, the rAIx of those in their 20 s was significantly lower than that of subjects in other age groups; on the other hand, the PSI of those in their 20 s was significantly higher than that of subjects in other age groups. These findings indicate that the rAIx and PSI are subject to age-dependent alterations, with sharper pulse waves appearing in younger individuals. In general, blood vessels become stiffer with age, so reflected waves in older people return more quickly and create more overlap with forward waves, causing blunter pulse waveforms^3^. These hemodynamics affect both the rAIx and PSI, which could be observed in the systolic phase. In addition, these findings are consistent with those of a prior study showing that components of systole could be principal components contributing to age-related factors of the radial pulse^8^. These sex and age differences could be observed more clearly through the morphology of the ensemble average pulse waveforms according to sex and age. The relationship between the change in the rAIx and PSI was investigated, and the predictive role of both indices could be explored though the waveform morphology.

There were significant associations between age and various indices. The rAIx and distance w used in previous studies showed similar positive correlations with age. In addition, the correlation between the EPA and age (r = 0.497) was slightly weaker than that between the rAIx and age, and VH showed no strong correlation with age (r = 0.284). However, the PSI calculated by combining the EPA and the VH showed the best correlation with age (r = − 0.550). Thus, the EPA, which is focused only near the peak point region, and the VH, which plays an additional role in considering the overall shape of the percussive wave, seem to complement each other. Because the PSI calculated by the robust algorithm showed a usefulness that could be applied to various waveform morphologies and showed a correlation with age at least as strong as that of the rAIx, the possibility of its becoming another indicator reflecting vascular aging, in addition to the rAIx, could be explored. To do this, simple regression analysis was performed, and the PSI showed a strong and significant negative correlation with arterial stiffness inferred by the rAIx (r = − 0.700), and an appropriate regression line could be estimated. Although the correlation between the rAIx and pulse apex angle derived by w has been observed in a previous study (r = 0.428)^24^, the correlation between the rAIx and PSI in this study showed a much higher value. Based on these relationships between the PSI and age and between the PSI and arterial stiffness, the PSI might be a candidate index to reflect vascular aging and to infer stiffness. In addition, because vascular stiffness is also linked to blood pressure, the relationship between PSI and age at identical levels of blood pressure was also examined^32,33^. PSI showed a decreasing pattern with aging in all three identical levels of blood pressure groups, and the group with lower SBP generally showed higher PSI values. Even though, there is relatively little data for people over 50 years old, nevertheless it was possible to roughly confirmed that PSI as a quantitative index of vascular aging was related to blood pressure.

The MLR models for the rAIx and PSI considering age and sex together showed a stronger correlation than the simple models considering only age. In addition, the MLR model for the PSI could be suggested to be slightly more accurate than the model for the rAIx. Furthermore, by analogy with β, it was found that age rather than sex had more influence on both rAIx and PSI. The results of rAIx are consistent with the findings of prior studies that age is the most powerful predictor of the rAIx compared to sex, height, HR and so on^10,30^.

The MLR analysis of arterial stiffness using the PSI, age, sex and HR showed that the four independent variables were significantly associated with stiffness, and the influence of the PSI on stiffness was found to be greater than that of any other variable, including age, which is known as one of the most important factors of stiffness. This means that the PSI contains more information regarding stiffness than age; of course, the age characteristic is also included in the PSI itself. Increasing HR causes a shift in the reflected wave from systole into diastole due to shortening of the ejection duration, which leads to a lower rAIx^34^. For this reason, HR was also associated with stiffness.

To evaluate the performances of the three simple regression models and an MLR model in this study, tenfold cross-validation was performed. When using the same age group as an independent variable, model 2 showed slightly better performance than model 1, as shown in Table 3. These results indicate that the model for the PSI can reflect vascular aging at least as much as the model for the rAIx. To investigate optimal fitting of the model for arterial stiffness inferred by the rAIx, the PSI alone was first considered in model 3, and four variables were all considered in model 4. Model 3, using the PSI, showed quite better performance than model 1, using age, which means that the PSI could be a more suitable index for reflecting stiffness than age. In addition, model 4 showed the highest performance among the tested models. Approximately 70% of the variance for stiffness could be accounted for by this model, and arterial stiffness could be approximated well from the PSI, age, sex and HR.

In general, the rAIx increases progressively with age up to 60 years and then plateaus, which might be attributable to impaired contractility^10,35^. In this study, quadratic relations were additionally considered in each regression model, but the performance of these models was not noticeably better than that of the linear models. This is thought to be because there were few data for subjects in their 60 s or older in this study. Therefore, more pulse data from elderly people are required to closely examine changes in the PSI in advanced age. In addition, this study did not consider the PWV, which has been widely used for reflecting arterial stiffness. Although the comparison between AIx and PWV has shown a significant association in prior studies^36,37^, detailed comparative analysis of the PSI, rAIx and normal PWV is required for a wider and more convincing generalization. And characteristics of the PSI by age based on hemodynamics or heart function should be also investigated^38^. According to our results, the larger the rAIx, the smaller the PSI tended to be, but it couldn’t always be asserted. The reasons of slightly different tendencies are thought to be that there are various factors affecting the shape of the waveform such as the amplitude or speed of reflected wave and cardiac ejection capacity. Furthermore, this study focused only on the sharpness of radial pulses. The sharpness quantification of aortic, carotid or brachial pulse waveforms and its clinical importance remain to be explored. Nevertheless, it is considered that there is a sufficient possibility that PSI can be used in arteries other than the radial artery. The PSI algorithm may need to be slightly modified due to differences in hemodynamic characteristics for each region, but the basic concept is not expected to be significantly different.

In this study, to complement the limitations of the rAIx, the PSI using a robust algorithm was proposed. The PSI could be uniformly applied to various pulse morphologies without having to consider the shape of the waveform in clinical practice, unlike the rAIx. In addition, this study confirmed that the PSI could be a quantitative index of vascular aging and has potential for use in inferring arterial stiffness with an advantage over the rAIx.

## Methods

### Experimental data

In this study, left radial artery pulse data recorded in a comfortable upright position from 528 subjects aged 20–84 years were analyzed. These data were collected as part of the Korea Institute of Oriental Medicine (KIOM) project: Development of pulse analysis system for personalized medicine by converging hemodynamics and pulse diagnostics. All 6 clinical trials over the 5 years were approved by the Institutional Review Board of the Kyung Hee University Korean Medicine Hospital: KOMCIRB-2014-70(KCT0002302); KOMCIRB-150622-HR-021(KCT0001604); KOMCIRB-150818-HR-030(KCT0001663); KOMCIRB-160620-HR-031(KCT0002070); and the Institutional Review Board of Gwangju Medical Center, Wonkwang University: 2015/8(KCT0001676); 2016/8-1(KCT0002147). The registration numbers of Korean Clinical Research Information Service are given in parentheses. All studies were conducted in accordance with the principles of the Declaration of Helsinki, and subjects signed written informed consent forms and agreed to the use of their pulse data for the study. Only normal control group subjects without any other medical history were used in this study.

### Measurements and signal processing

Pulse signals were measured using a pulse tonometric device (KIOM-PAS ver. 2.0, Republic of Korea) developed by KIOM. The device has been widely used in many clinical studies and several reliable variables calculated using this device have been found to have clinical significances^23,39–41^. The reliability and safety of the device were confirmed based on clinical good manufacturing practices by the Ministry of Food and Drug Safety of Korea, and the electromechanical stability of the device was certified based on the IEC 60601-1 2nd edition. The six-channel piezo-resistive sensor arrayed in a row was used to obtain the pulse signal and the signal from the channel with the largest amplitude was fi-nally selected to analysis. The sensing element has dimensions of 1 × 1 square millimeters (C33, EPCOS, Germany). The continuously evolving tonometric mechanism with a sampling rate of 1000 Hz was applied to the device, and a 60-s pulse series under the same applied pressure was used for the ensemble average pulse analysis^39^.

The pulse signal was smoothed using the weighted Handerson’s moving average algorithm, and noise from 0.005 to 30 Hz was minimized by applying a Butterworth filter. Baseline correction was performed by third-order spline fitting and spline interpolation, and ITM was applied to extract the starting point of each pulse wave^42^. Each separated pulse was truncated to the minimum size among the intervals from each starting point to the next starting point. After amplitude normalization, an ensemble average pulse was obtained from a well-refined pulse series. Since individual average pulse signals always have unequal lengths, the pulse length was set at the same interval of 0.8 s by applying resampling method^3,34,43^. The above signal processing and variable calculations mentioned later were performed on all pulse waveforms using LabVIEW 2016 (National Instruments, USA).

### rAIx

The algorithm based on the numerical fourth derivative pulse waveform was applied to calculate the rAIx with the following equation: (late systolic blood pressure/early systolic blood pressure) × 100 (%)^9^. Because the rAIx decreases linearly with increasing heart rate due to an alteration in the relative timing of the reflected pressure wave caused by a reduction in the ejection duration^34,44^, the rAIx was corrected for a HR of 75 beats per minute (bpm)^45^. The PSI proposed in this study can be calculated from any shape of the waveform. However, because a comparative analysis with the previously used rAIx is required in this study, waveforms where the amplitude of the tidal wave did not appear clearly or was obscure were excluded. About 4.5% (24 out of 528 waveforms) of waveforms were found to be difficult to calculate rAIx accurately, and 504 waveforms that could be used to calculate the rAIx clearly were used for analysis^10^.

### Feature extraction and calculation of the pulse sharpness index (PSI)

The ITM mentioned above is the most well-known algorithm with the best performance in extracting the starting point of the pulse waveform^46^. In this study, the idea of the ITM was converted and applied to the peak point of the percussive wave rather than the starting point of the ensemble average pulse, and feature points for pulse sharpness were detected by this switching of the ITM concept.

**Figure 3:**
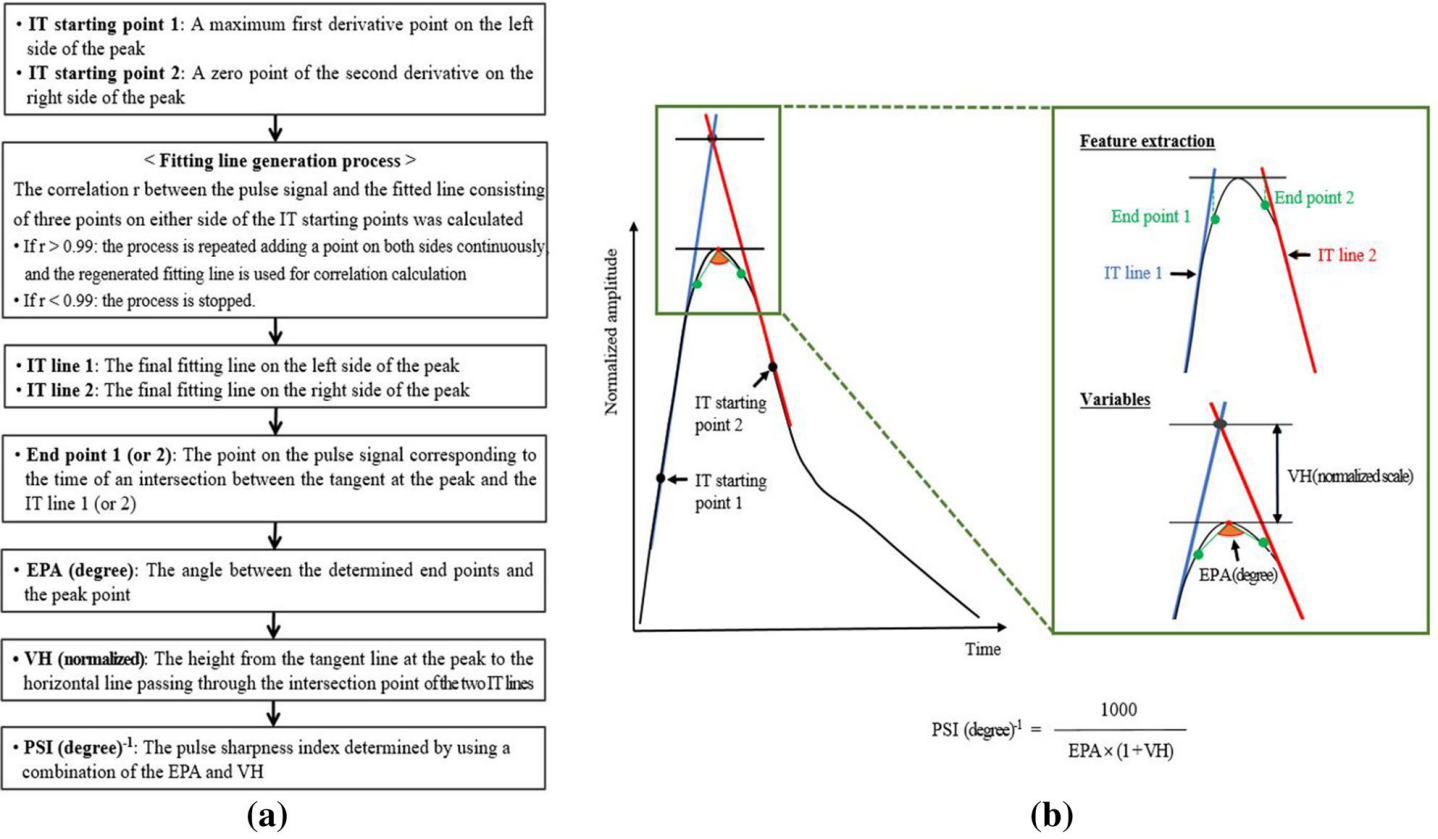
(**a**) Flowchart of feature extraction and calculation of the PSI, and (**b**) it’s graphical descriptions. *IT* intersecting tangent, *EPA* end point angle, *VH* virtual height, *PSI* pulse sharpness index.

First, the correlation coefficient between the average pulse signal and the fitted line consisting of three points on either side of the intersecting tangent (IT) starting point 1 and 2 was calculated. If the correlation was greater than 0.99, points were then added on both sides for regenerating fitted line of five points. This process was repeated adding a point on both sides continuously until the correlation was less than 0.99. Then, if the correlation was less than 0.99, the fitting line generation process was stopped and the final regenerated fitting line becomes IT line 1 or 2. After that, the point on the pulse signal corresponding to the time of an intersection between the tangent at the peak and the IT line 1 or 2 was extracted as end point 1 or 2. A flowchart of the feature extraction and calculation of the EPA, VH and PSI is presented with graphical descriptions in Fig. 3. The EPA could generally reflect the pulse sharpness well based on the solid standard method with feature points, but it might be a local variable that focuses only near the peak point regions. Another variable of VH could consider the overall shape of the percussive wave. Finally, the PSI was determined by using a combination of the EPA and the VH, and it was converted to a natural number by simply multiplying by 1000 as follows:

1 $${PSI \left(degree \right)}^{-1}=\frac{1000}{EPA \times \left(1+VH\right)}$$

A sharp pulse waveform yields a high PSI, whereas a blunt pulse waveform yields a low PSI. Differences in the pulse sharpness inferred by the EPA, VH and PSI were compared with the limitations of the two single indicators. The PSI could reflect the pulse sharpness intuitively, unlike the EPA and VH, and details are provided in Supplementary Fig. S3. The PSI could be calculated for all waveforms regardless of the shape of the pulse waveforms. Additionally, the distance w between the points on both sides of the waveform at 2/3 the pulse height was determined to infer the pulse apex angle, as previously described^24–27^.

### Statistical method

The demographic and baseline characteristics including vital signs of the 504 subjects by sex were summarized. All subjects were separated into four groups based on age, i.e., those in their 20 s, 30 s, 40 s and 50 s or older (N = 147, 111, 180, and 66, respectively), and all statistical methods were applied for the rAIx and PSI.

First, to compare the mean differences between the men and women in each age group, independent t-tests were applied. In addition, the mean differences by age group for each sex were analyzed by one-way ANOVA with Duncan’s post hoc test. If Levene’s test for equal variance was violated, Welch’s robust ANOVA with Dunnett’s T3 post hoc test was applied. The level of significance was set to P < 0.05. Representative ensemble average pulse waveforms by sex and age group were generated to observe the typical patterns of waveforms and age-related changes.

Next, correlation analyses of age with the rAIx, w and the PSI were performed with the r, and linear regression analyses to explore the effect of the PSI on arterial stiffness were performed. At this time, in order to further examine the relationship between blood pressure and other variables, pulse data were divided into three groups according to recorded SBP at the index examination: group 1, < 120 mmHg; group 2, 120 to 139 mmHg; group 3, 140 to 159 mmHg^32^. In addition, MLR analyses of age and sex with respect to the rAIx following previous studies and with respect to the PSI to explore age-related characteristics were performed. Furthermore, combinations of the PSI, age, sex and HR were applied to determine the best regression model. Because the age distribution of our data was exponential with relatively little data for the elderly, linear regression using logarithm of the age was performed^47,48^. The results were described by the standardized regression coefficient (β), and a P value of < 0.05 was considered statistically significant. The cross-validated R^2^, RMSE and MAE after standardizing variables during tenfold cross-validation were used to measure the constructed regression model performance. For reference, in simple linear regression model 1, rAIx is expressed as an index name in order to examine the relation between age and the rAIx variable itself. On the other hand, in multiple linear regression models 3 and 4, arterial stiffness inferred by rAIx is expressed as an index name to examine whether the newly proposed PSI has relevance to arterial stiffness, and for direct interpretation. Mean difference analyses were performed using IBM SPSS statistics 20, and pulse waveform analyses and regression analyses with validation were performed using MATLAB R2018b.

## Supplementary Information

Supplementary Information.
