# Supplementary material for: Pulse sharpness as a quantitative index of vascular aging
Source: Sci Rep. 2021 Oct 6;11:19895. doi: 10.1038/s41598-021-99315-8 (PMC8494761; doi:10.1038/s41598-021-99315-8)
Supplement: Supplementary file 1 — Supplementary Information. [file 41598_2021_99315_MOESM1_ESM.docx]

**Pulse sharpness as a quantitative index of vascular aging**

Jang-Han Bae^1^, Young Ju Jeon^1*^

^1^ Future Medicine Division, Korea Institute of Oriental Medicine, 1672 Yuseong-daero, Yuseong-gu, Daejeon, Republic of Korea.

Correspondence and requests for materials should be addressed to Y.J.J. (email: jyj92@kiom.re.kr)


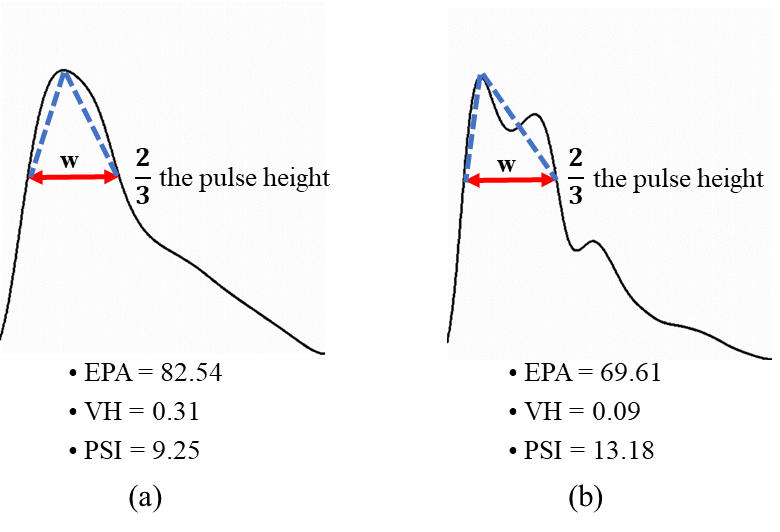


Supplementary Figure S1. Comparison of the pulse sharpness index (PSI) according to pulse waveform samples with differences in pulse sharpness. The PSI could be uniformly applied to various pulse morphologies including waveforms that are difficult or too obscure to use in calculating the radial augmentation index (rAIx) or pulse apex angle using w accurately. W was indicated to intuitively show the limitations of 2/3 the pulse height method.

(a) Radial pulse waveform that is obscure to determine the rAIx clearly and for which the sharpness angle based on w is underestimated; (b) Radial pulse waveform for which the sharpness angle based on w is overestimated;


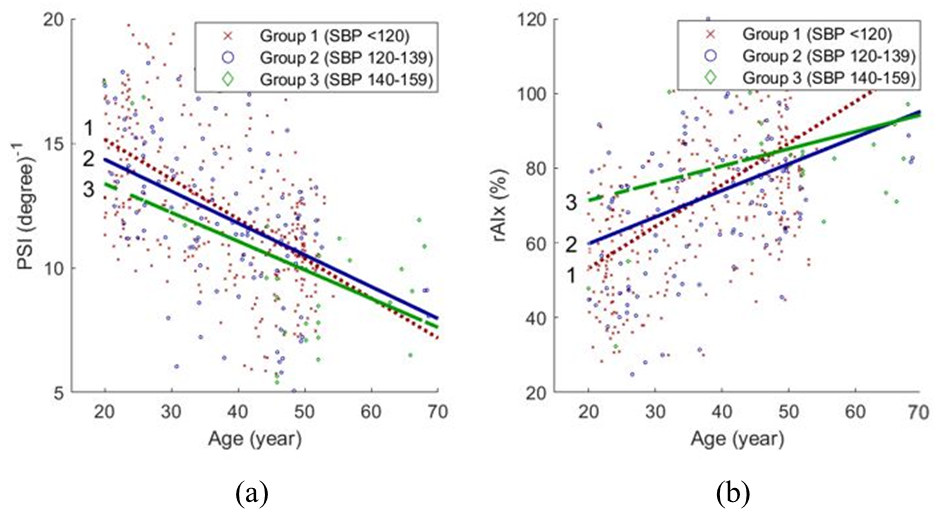


**Supplementary Figure S2.** (a) Scatterplots of relationship and the regression line between age and the pulse sharpness index (PSI). (b) Scatterplots of relationship and the regression line between age and the radial augmentation index (rAIx). Pulse data were divided into three groups according to recorded systolic blood pressure (SBP) at the index examination: group 1, <120 mm Hg; group 2, 120 to 139 mm Hg; group 3, 140 to 159 mm Hg.


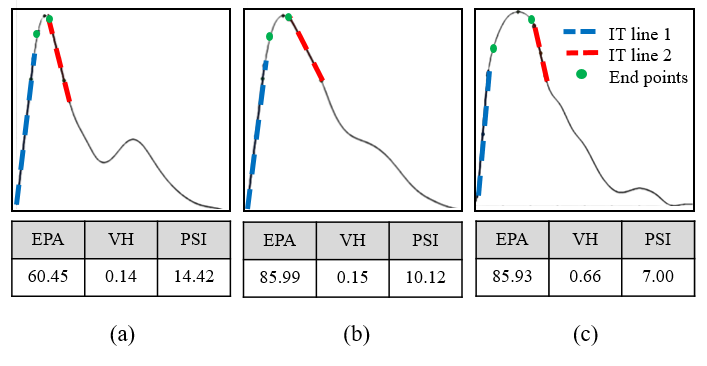


**Supplementary Figure S3.** The three types of pulse waveforms with differences in pulse sharpness and a comparison of the calculated end point angle (EPA), virtual height (VH) and pulse sharpness index (PSI) values. IT, intersecting tangent.

• Although waveform (a) is sharper than waveform (b), the VH of each is similar. If the slope of IT line 2 is gentle, even though the pulse waveform is blunt, the limit of the VH, whose value is not very large, is represented.

• Although waveform (b) is sharper than waveform (c) near the vertex, the EPA of each is similar. If an uncommon slope appears in the peak region, the limit of the EPA, which is only considered near the peak region, is represented.

• The PSI calculated using the EPA and the VH could reflect the pulse sharpness intuitively, as expected, with the highest value for waveform (a) and the lowest value for waveform (c).
